# Mutations in the CDS and promoter of *BjuA*0*7*.*CLV1* cause a multilocular trait in *Brassica juncea*

**DOI:** 10.1038/s41598-018-23636-4

**Published:** 2018-03-28

**Authors:** Lu Xiao, Xin Li, Fei Liu, Zhi Zhao, Liang Xu, Cuiping Chen, Yanhua Wang, Guoxia Shang, Dezhi Du

**Affiliations:** 1 State Key Laboratory of Plateau Ecology and Agriculture of Qinghai University, Key Laboratory of Qinghai Province for Spring Rapeseed Genetic Improvement, The Qinghai Research Branch of the National Rapeseed Genetic Improvement Center, Qinghai Academy of Agricultural and Forestry Sciences, Qinghai University, Xining, 810016 China; 2 National Key Lab of Crop Genetic Improvement, National Center of Rapeseed Improvement in Wuhan, Huazhong Agricultural University, Wuhan, 430070 P.R. China

## Abstract

Multilocular trait has recently attracted considerable attention for its potential to increase yield. Our previous studies indicated that two genes (*Bjln1* and *Bjln2*) are responsible for multilocular siliques in *Brassica juncea* and the *Bjln1* gene has been delimited to a 208-kb region. In present study, the *Bjln1* gene was successfully isolated using the map-based cloning method. Complementation test indicated that the *BjuA07*.*CLV1* (equivalent to *BjLn1*) could rescue the multilocular phenotype and generate bilocular siliques. Two amino acids changes at positions 28 and 63 in BjuA07.clv1 as well as a 702-bp deletion in its promoter have been proved to affect the carpel numbers. Microscopic analyses suggested that *BjuA07*.*CLV1* is involved in the maintenance of shoot and floral meristem size. The expression level of *BjuA07*.*clv1* was significantly reduced in the SAM. Furthermore, *WUS*, *CLV2*, *CLV3*, *RPK2* and *POL*, key genes in the *CLV/WUS* signal pathway, showed lower expression level in the multilocular plants. These data suggest that the mutations in the CDS and promoter of *BjuA07*.*clv1* reduced its function and expression level, which disturbed *CLV/WUS* signal pathway, thereby leading to the enlargement of the shoot and floral meristem and resulting in the multilocular siliques.

## Introduction

*Brassica juncea* (*B*. *juncea*, AABB, 4n = 36) is cultivated as a main oilseed crop in China. It has many desirable agronomic traits such as drought resistance, barrenness tolerance, and disease and pest resistance. It has been widely used as a valuable resource for the genetic improvement of *Brassica napus*. However, the lower yield of *B*. *juncea* largely limited its usage in breeding.

Multilocular trait, which was firstly detected in *B*. *juncea*, has great potential to increase yield. Unlike the ordinary bilocular *B*. *juncea*, multilocular *B*. *juncea* produce siliques with multiple locules, and the yield per plant is significantly higher than that of bilocular *B*. *juncea* in the same genetic background^1^.

Generally, oilseed siliques develop from a gynoecium consisting of two carpels that grow and combine with each other to form a locule, i.e., gynoecium (or ovary). After the development of the ovary, the medial ridges grow towards each other and disappear to form a false septum, dividing the ovary into two locules^2^. The ovules grow in the locule of the developed ovary.

Carpels originate from the floral meristem (FM) which is formed on the flanks of an indeterminate shoot apical meristem (SAM) when the plants enter the reproductive growth period in oilseeds. In higher plants, continuous organogenesis is accomplished by SAM, a collection of self-dividing cells that generate descendant cells during differentiation^2,3^. The SAM of angiosperms is usually classified into two regions: a central zone (CZ) and a peripheral zone (PZ). The CZ, a cell cluster in a pyramidal form on the tip of SAM, serves as a fundamental source of all shoot cells, whereas the PZ surrounds the central zone and facilitates the differentiation and initiation of organs^4–6^.

The dimension of the CZ is precisely regulated by the CLAVATA (CLV) signaling pathway^2,6,7^. *CLV* genes interact with the *WUSCHEL*(*WUS*) gene and establish a negative feedback loop between the stem cells and the organising centre below, thereby maintaining the stem cell population in the SAM^4,8,9^. The *BARELY ANY MERISTEM 1* (*BAM1*) gene and *BARELY ANY MERISTEM 2* (*BAM2*) gene, encoding receptor kinase-like proteins with the role opposite to that of CLV1, regulate SAM specification by complex interaction with the CLV pathway^10,11^. Mutations in the genes cause defects in SAM and/or FM development.

Recently, a study indicated that a point mutation (C-to-T) in exon 3 of *BrCLV3*, was found to cause an amino acid change of Pro-to-Leu in the mutant *ml4*, resulting in a multilocular trait in *B*. *rapa*^12^. Xu *et al*.^13^ reported that trilocular phenotype in *B*. *juncea* was caused by the insertion of a copia-LTR retrotransposable element in the coding region of *BjMc1*.

Duoshi is a *B*. *juncea* cultivar originated from the Qinghai-Tibetan plateau and it produces siliques with 4 locules^1^. Genetic studies indicated that two recessive genes (*Bjln1* and *Bjln2*) were responsible for the multilocular phenotype in Duoshi^14^. Previous mapping study has delimited *Bjln1* to a 2.4 cM interval^14^. In the present study, *BjLn1* gene was successfully isolated using map-based cloning based on the collinearity between *B*. *juncea* and *B*. *rapa*, along with the *Arabidopsis* genome. We reported that the reduced function and expression level of *BjuA07*.*clv1* (equivalent to *BjLn1*) caused the multilocular trait in Duoshi.

## Results

### Delimitation of *BjLn1* to an 85-kb syntenic region

**Figure 1:**
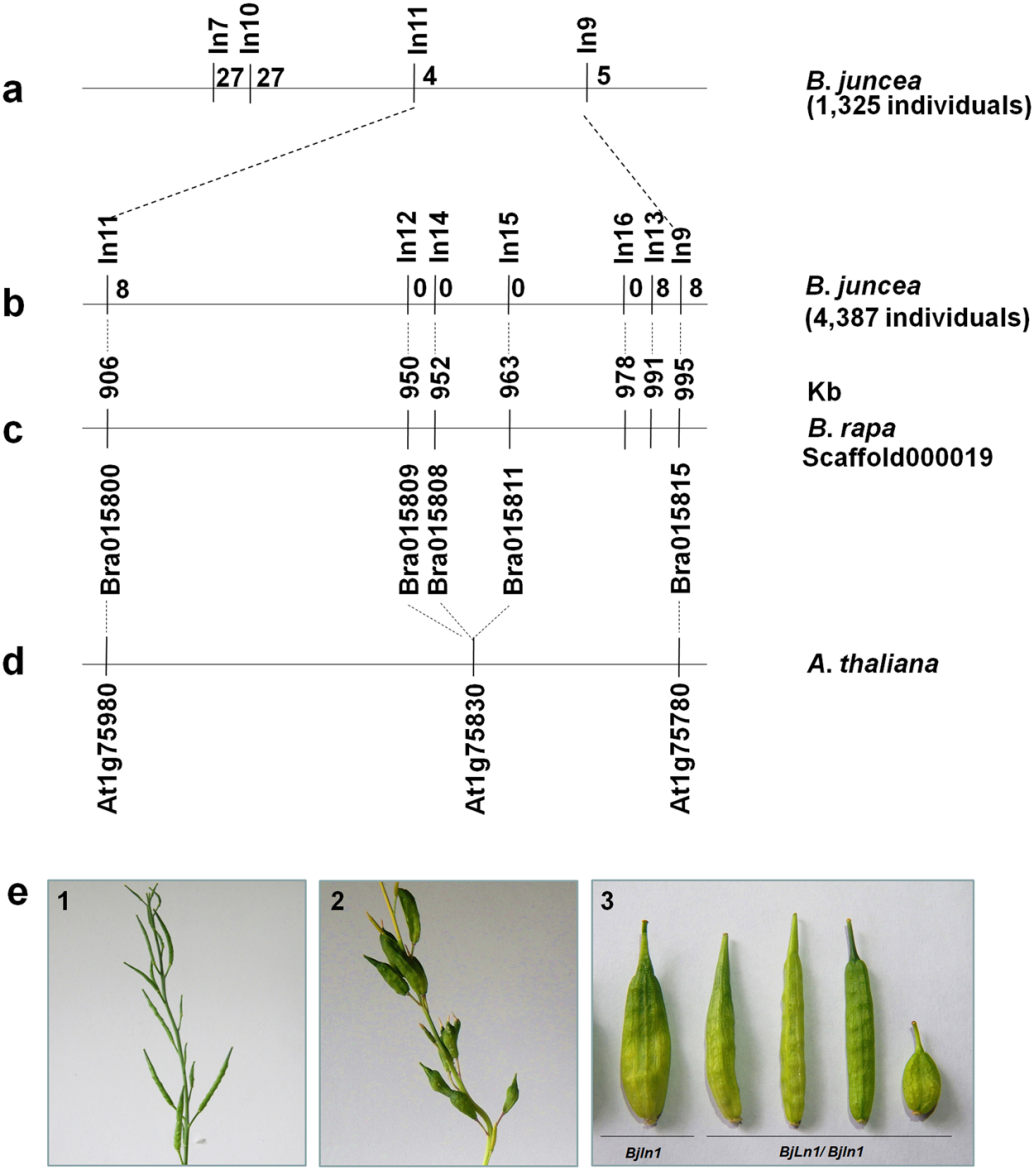
Physical mapping of the *BjLn1* gene and phenotypes obtained from genetic complementation test. **(a–d)** The Arabic numerals near the position of the marker represent the recombinant number of the markers with the *Bjln1* gene. Positional cloning narrowed the *BjLn1* locus to an 85-Kb region on the A07 chromosome between markers ln11 and ln13. **(e)** Phenotypes obtained in the genetic complementation test. (1) Representative siliques of a bilocular T_0_ transgenic plant in the Duoshi background. (2) Representative siliques of a multilocular non-transgenic plant. (3) Comparison of the single silique in the non-transgenic Duoshi plant (the left one, genotype: *Bjln1*) and the normal-shaped bilocular silique of T_0_ transgenic plant (the second to the fifth silique, genotype: *BjLn1*/*Bjln1*) in the *Bjln1* background.

Previous studies have shown that the *BjLn1* gene was localised on the A07 linkage group of *B*. *rapa*, and the region spanning the target gene has perfect collinearity with *A*. *thaliana* chromosome 1^14^. Based on the putative *A*. *thaliana* syntenic region, 50 intron polymorphism (IP) primers^15^ were designed for more precisely mapping of the *BjLn1* gene. Two polymorphism IP markers (ln10 and ln11) were found and mapped relative to *BjLn1* in a previous population of 1,325 BC3 plants. The markers ln10 and ln11, on the same side as ln7, were 2.0 cM and 0.3 cM, away from *BjLn1*, respectively. Therefore, *BjLn1* was genetically located at an interval of 0.7 cM between the markers ln11 and ln9 (Fig. 1a).

The BC3 population of 4,387 individuals derived from the cross of Duoshi (multilocular parent) × Tayou2 (bilocular parent) was developed to further delimitate the *BjLn1* locus. Two closest flanking markers (ln11 and ln9) were used to detect recombinant events in this BC3 population and eight recombinants for each marker were detected. Further, simple sequence repeat (SSR) markers were developed based on the *B*. *rapa* sequence in the delimited region. Five SSR markers, designated as ln12–ln16, were identified and subsequently used to screen the 16 recombinants. Eight recombinants were detected between ln13 and *BjLn1*, whereas no recombinants were detected between the remaining 4 SSR markers and *BjLn1*. Hence, the *BjLn1* gene was eventually narrowed down to a genetic region between markers ln11 and ln13, corresponding to an approximately 85-kb syntenic interval of A07 in the *B*. *rapa* genome (Fig. 1b). Searching these marker sequences against National Center for Biotechnology Information (NCBI, http://www.ncbi.nlm.nih.gov/) revealed that 2 IP and 5 SSR markers had sequence homology with the bottom of chromosome 1 from *A*. *thaliana* and chromosome A07 from *B*. *rapa* (Fig. 1c–d). The microsynteny analysis of the *BjLn1* gene revealed a candidate homologous region containing 16 genes in *B*. *rapa* (*Bra015800*–*Bra015815*; Table 1), corresponding to *A*. *thaliana* homologues *At1g75780* to *At1g75980* (22 genes; Table 1). Of the 22 *Arabidopsis* genes, *CLAVATA1* (*CLV1*, *At1g75820*) plays a key role in SAM and FM identity maintenance in *A*. *thaliana*; T-DNA mutation in *CLV1* causes excess stem cell accumulation in the centre of the SAM, resulting in enlarged SAM and FM^16^. An increase in FM size is mainly reflected in increased flower organ numbers, especially carpels^2,17^. Thus, we speculated that the homologous *CLV1* gene (*BjuA07*.*CLV1*) in *B*. *juncea* is most likely the candidate gene of *BjLn1*.

**Table 1:** The *Brassicarapa* genes in the target region and their best hits to *Arabidopsis thaliana* genes using BlastN.

*B*. *rapa* genes	*Arabidopsis* homologues	*E* value	*Arabidopsis* annotations (molecular function)
Bra015815	AT1G75780	0	beta tubulin gene; TUB1; GTP binding/GTPase activity/structural constituent of cytoskeleton
Bra015814	AT1G75800	e-107	unknown protein
Bra015813	AT1G75810	9e-55	unknown protein
Bra015812	AT1G75820	0	CLV1 (CLAVATA1); ATP binding, kinase activity, protein binding, protein self-association, protein serine/threonine kinase activity, receptor serine/threonine kinase binding
Bra015808	AT1G75830	3e-09 (2e-36, 4e-40)	unknown protein
Bra015807	AT1G75840	e-39	ROP4; GTP binding/GTPase activity/protein binding
Bra015806	AT1G75850	e-98	protein transporter activity
Bra015805	AT1G75860	2e-17	unknown protein
Bra015804	AT1G75880	e-71	lipase activity, transferase activity, transferring acyl groups
Bra015803	AT1G75890	e-53	lipase activity, transferase activity, transferring acyl groups
Bra015802	AT1G75900	6e-80	lipase activity, transferase activity, transferring acyl groups
Bra015801	AT1G75950	7e-50	protein binding, ubiquitin-protein transferase activity
Bra015800	AT1G75980	8e-38	unknown protein

### *BjuA07*.*CLV1* decreases locule number in *B*. *juncea* Duoshi

In order to verify our speculation, complementation test was performed. The complementation construct based on the binary vector pCAMBIA2300 encompassing the 1,945-bp upstream region; the 3,044-bp ATG to TGA region; and the 376-bp downstream region of the candidate homologous *CLV1* gene was transformed into the multilocular parental line (Duoshi). The untransformed control plants produced 100% multilocular siliques. More than 50 transgenic positive plants (T0) were obtained and scored for the locule number of the siliques during the mature period in the greenhouse. Overall, 25 plants showed recovery of some bilocular siliques, and six plants exhibited a higher bilocular silique percentage (>80% siliques; Fig. 1e, Supplementary Fig. S1). The six plants were self-pollinated. The resulting T1 progenies randomly selected from one T0 plant were planted in a greenhouse. Perfect co-segregation between the transgenic plants and phenotype was observed (Supplementary Fig. S2), which suggesting the recovery of the T1 plants could be stably co-transmitted and co-segregated with the introduced DNA. Therefore, the candidate homologous *CLV1* gene, *BjuA07*.*CLV1*, was experimentally confirmed to correspond to the *BjLn1* gene, and *Bjln1* was named as *BjuA07*.*clv1*.

### *BjuA07*.*CLV1* affects seeds rows

**Figure 2:**
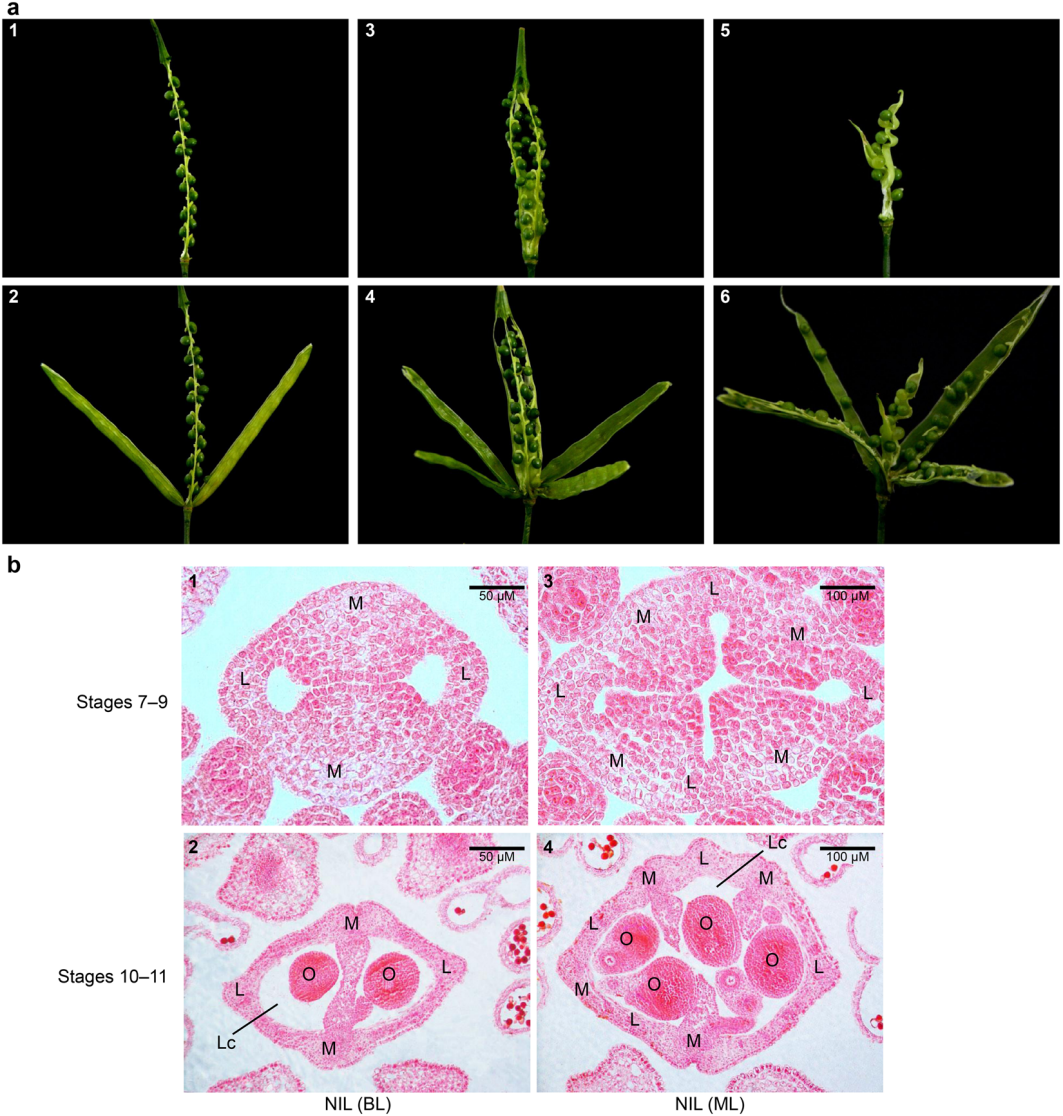
Comparison of silique phenotype between multilocular and bilocular plants in BC3F5 generation. (**a)** An anatomical diagram of a bilocular and a multilocular silique in BC3F5 plants. (1–2) A normal *B*. *juncea* silique with two valves (2), or with both the valves removed (1), and two rows of seeds in it. (3–4) A multilocular silique with four valves (4), or the four valves have been removed (3), and four rows of seeds in it. (5–6) A multilocular silique possessing an additional pistil in the silique (6), an additional gynoecium inside the silique that has been peeled, and seeds are in and around the gynoecium (5). (**b)** Cross-sections of the gynoecium from the bilocular and multilocular plants at different stages of development. (1) Bilocular plant of BC3F5, stages 7–9. (2) Bilocular plant of BC3F5, stages 10–11. (3) Multilocular plant of BC3F5, stages 7–9. (4) Multilocular plant of BC3F5, stages 10–11. M, medial region; L, lateral region; O, ovule; Lc, locule.

We subsequently compared the seeds per silique between bilocular and multilocular plants in BC3F5. The results indicated that the seeds per silique in multilocular plants (28 ± 3.09) were significantly higher than those in bilocular ones (18 ± 1.84) (t = 16.7, p < 0.01). Therefore, the seeds per silique in multilocular plants in the same genetic background significantly increased. After the siliques of the oilseeds were peeled, we found that the multilocular siliques usually had four fruit valves and contained four rows of seeds, whereas bilocular siliques usually had only two fruit valves and two rows of seeds (Fig. 2a1–4). Each row of seeds was generated from the placental tissue positioned at the junctions between the fruit valves and septa (Fig. 2a). Therefore, the number of fruit valves, which originate from the carpels, determined the number of seed rows in the oilseed siliques. The cross-section of the siliques further confirmed that multilocular siliques had four locules, each with one row of seeds, whereas bilocular siliques had two locules and contained two rows of seeds (Fig. 2b). An endogenous gynoecium that produced seeds was found inside the siliques of multilocular plants (Fig. 2a5–6). Therefore, the presence of multiple carpels results in the occurrence of multiple seed rows, and multiple carpels and endogenous gynoecium contributed to the increased seeds per silique in multilocular plants.

### *BjuA07*.*CLV1* affects the dimension of SAM and FM

**Figure 3:**
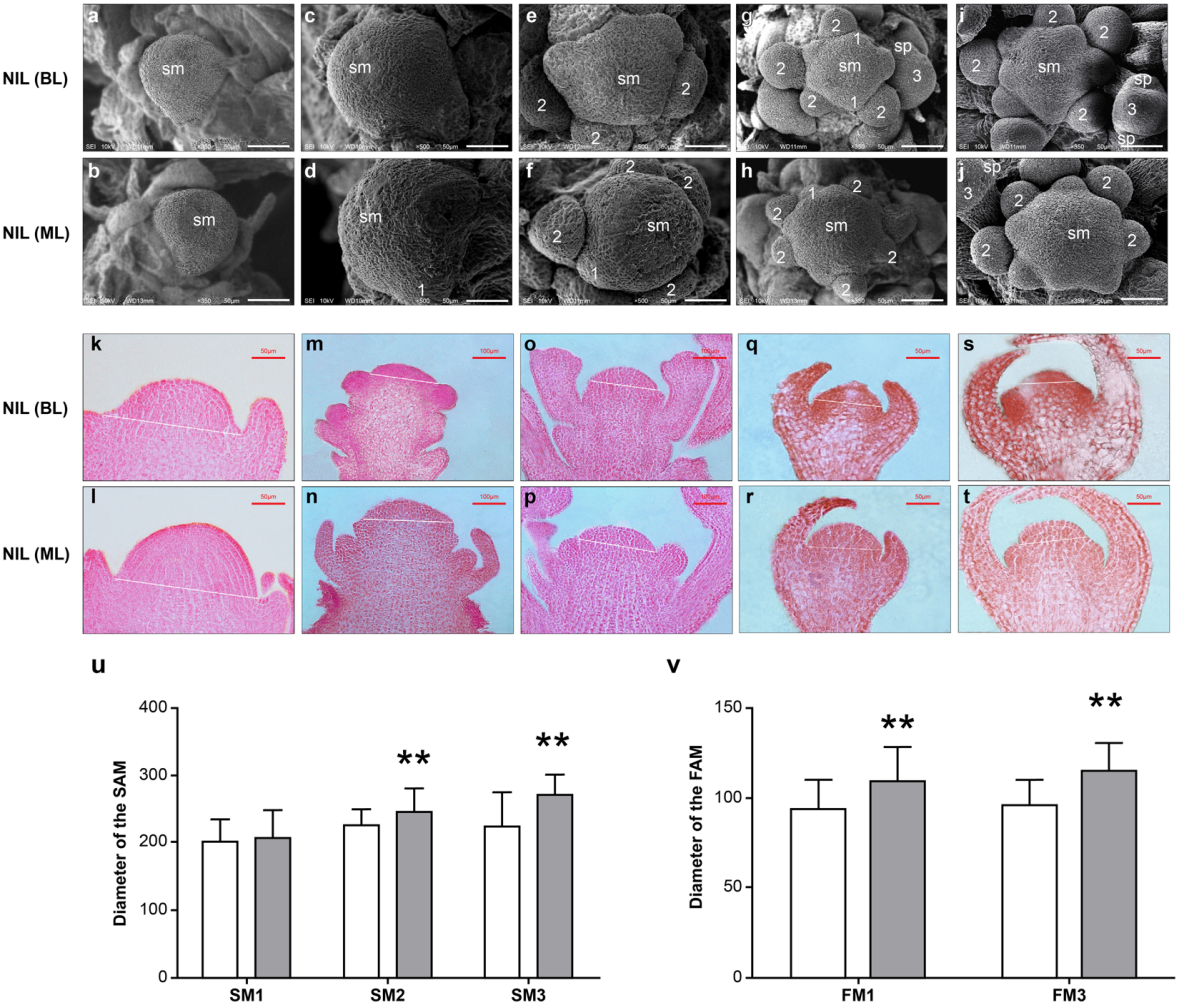
The phenotype and longitudinal sections of shoot apical meristem (SAM) and floral meristem (FM). (**a**–**j**) The phenotype of SAM. The SAM of a bilocular (**a**) and multilocular plant (**b**). No inflorescence meristems have formed. No visible morphological differences were noted between the SAM from bilocular and multilocular plants. The SAM of bilocular (**c**) and multilocular plants (**d**) was surrounded by individual FM, at flower development stage 1. The SAM of near-isogenic lines (NIL) is enlarged and remains larger in multilocular plants than in bilocular plants. The SAM of bilocular (**e**) and multilocular plant (**f**) was surrounded by few FMs. The flower primordium separated from the SAM, and flower development reached stage 2. The SAM of bilocular (**g**) and multilocular plants (**h**) was surrounded by many FM, and the flower development reached stage 3 with the sepal primordium visible. The SAM of bilocular (**i**) and multilocular plant (**j**) was surrounded by abundant FM, and the floral primordia constantly elongated and exceeded the shoot apex. White lines indicate boundaries for width measurement. sm, shoot meristem; sp, sepal. (**k**–**t**) Longitudinal sections of SAM and FM. Young SAM with no FM formed in bilocular (**k**) and multilocular plants (**l**). (**m**,**n**) The SAM with many surrounding FMs in bilocular (**m**) and multilocular plants (**n**). The SAM with abundant surrounding FMs in bilocular (**o**) and multilocular plants (**p**), and some floral primordia constantly elongated and exceeded the height of the shoot apex. The flowers in bilocular (**q**) and multilocular plants (**r**) at stage 4; the sepal primordia grow to overlie the primordium. The flowers in bilocular (**s**) and multilocular plants (**t**) at stage 5; petal primordia appear. (**u-v**) Longitudinal section comparisons of SAM and FM diameter in bilocular and multilocular plants. u, the diameter of the seedling SAM (left, SM1) in bilocular and multilocular plants; the inflorescence SAM with many FMs around it (middle, SM2) and with abundant FMs around it (right, SM3) in bilocular and multilocular plants; v, diameter comparison of flowers in bilocular and multilocular plants at stages 4 (left, FM1) and 5 (right, FM3). **P = 0.01.

To understand how *BjuA07*.*clv1* caused the increase in carpel number, we investigated the SAM and FM. After comparing the shoot meristems between homozygous bilocular plants and multilocular ones in BC3F5 of the same developmental stage, we found that the SAM of multilocular BC3F5 plants was larger than that of bilocular ones during the flower bud differentiation stage (Fig. 3c–j). The diameter of the SAM from multilocular plants was significantly greater than that of the SAM from bilocular plants during the middle and late stages of flower bud differentiation (Fig. 3m–p,u). Furthermore, the size of floral meristems of multilocular BC3F5 plants was significantly greater than that of floral meristems of homozygous bilocular plants (Fig. 3q–t,v).

### Amino acid variations at positions 28 and 63 of BjuA07.CLV1 affected the number of carpel

**Figure 4:**
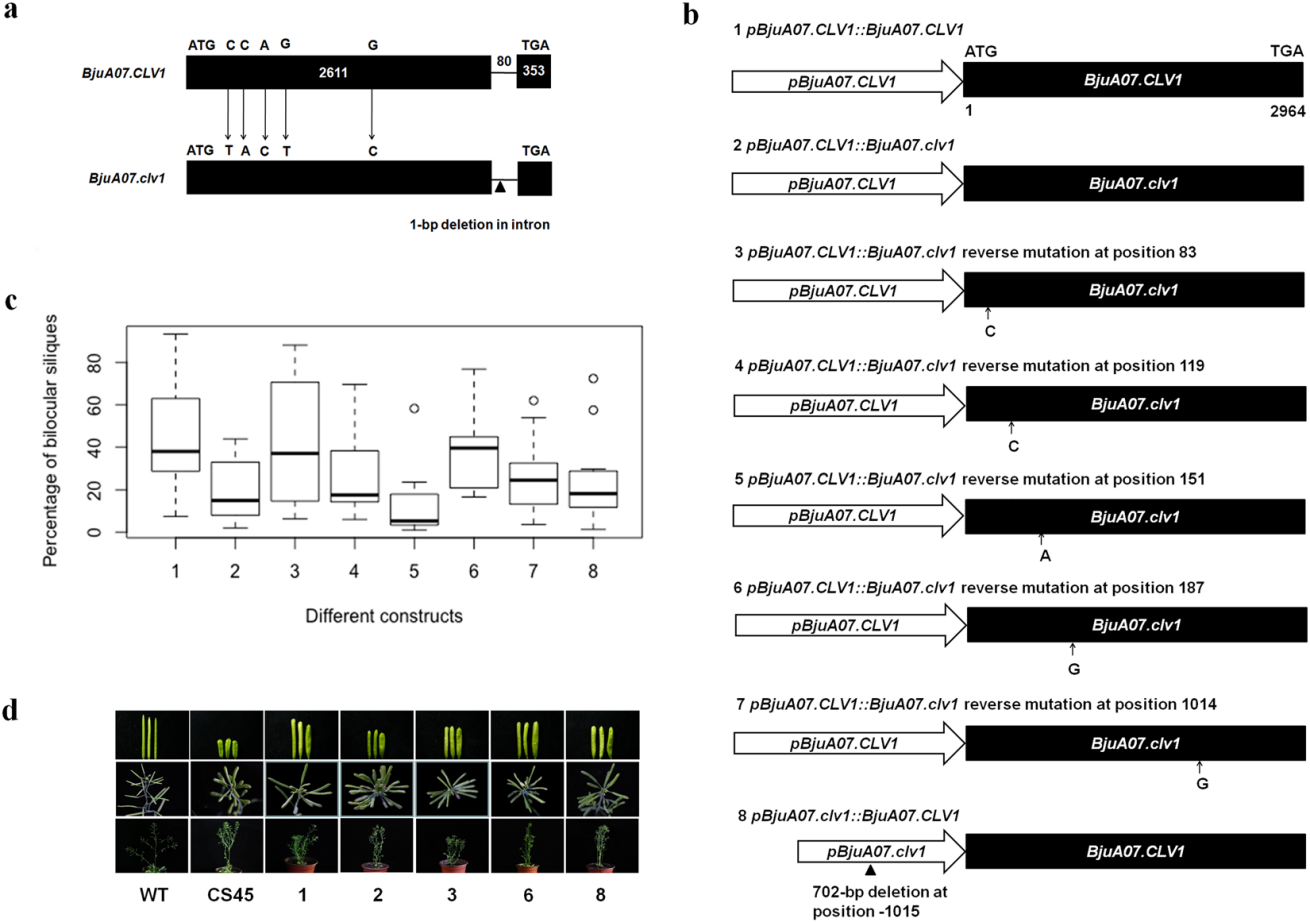
Identification of the sequence variations causing the changes of carpel numbers. (**a**) Gene structure of *BjuA07*.*CLV1* from Tayou2 and *BjuA07*.*clv1* from Duoshi in *B*. *juncea*. Black boxes represent the exons, and the black lines the introns. The initiation codon (ATG) and stop codon (TAG) are shown in the figure. The numbers in the black box and above the black linerepresent the nucleotides of the exons and intron, respectively. The arrows indicate five single nucleotide substitutions, resulting in amino acid changes. The individual black triangle represents deletion. (**b**) The schematic representation of the constructs synthesized by GENEWIZ Inc. (China), which was introduced to pCAMBIA2300, and then transformed the Arabidopsis mutant CS45. The white arrow represents the promoter, *pBjuA07*.*CLV1* and *pBjuA07*.*clv1* in it represents the promoter of the *BjuA07*.*CLV* and *BjuA07*.*clv1* gene. The black box represents CDS region, *BjuA07*.*CLV1* and *BjuA07*.*clv1* in it represents the different CDS from the *BjuA07*.*CLV* and *BjuA07*.*clv1* gene. The small black arrow and the nucleotide below represent the substitution position and the introduced base. The individual black triangle represents deletion. (**c**) Percentage of bilocular silique. The constructs of 1–8 is same as that in (**b**) of Fig. 4. (**d**) The T1 phenotype obtained in the genetic complementation test. The rows from top to bottom show the obtained phenotype of siliques, branch and the individual plant. The constructs used in the lines from left to right are 1, 2, 3, 6 and 8, respectively, which are same as that in (**b**) of Fig. 4.

To determine the genetic variation that causes the changes of carpel numbers, the genomic DNA and cDNA sequences of *BjuA07*.*CLV1* and *BjuA07*.*clv1* were isolated, respectively. Sequence comparison revealed that the *BjuA07*.*CLV1*/*BjuA07*.*clv1* contains two exons and one intron (Fig. 4a, Supplementary Fig. S3). The coding sequence of *BjuA07*.*CLV1* as well as *BjuA07*.*clv1* is 2964 bp, predicted to encode a polypeptide of 987 amino acids. Compared with the CDS of *BjuA07*.*CLV1*, *BjuA07*.*clv1* has fifty-nine single nucleotide polymorphisms (SNPs) (Supplementary Fig. S3). wherein, five SNPs at positions 83, 119, 151, 187 and 1014 caused amino acids changes. The proline, Threonine, asparagine, alanine and glutamate residues at positions 28, 40, 51, 63 and 338 of BjuA07.CLV1 were substituted by leucine, asparagine, histidine, serine and aspartic acid residues of BjuA07.clv1 (Supplemental Fig. S4).

In order to detect whether the five SNPs causing amino acid changes in *BjuA07*.*clv1* influence its function, the *pBjuA07*.*CLV1::BjuA07*.*CLV1* and *pBjuA07*.*CLV1::BjuA07*.*clv1* constructs were cloned in pCAMBIA2300, and transformed *A*. *thaliana* CS45 mutant (*clv1-1*, which produce the siliques with four carpels) (Fig. 4b). The *pBjuA07*.*CLV1::BjuA07*.*CLV1* transgenic plants displayed a recovery of bilocular siliques, and the average rescue rate was 45.5% (range: 7.5–93.3%). While the transgenic plants of *pBjuA07*.*CLV1::BjuA07*.*clv1* exhibited recovery with the average percentage of bilocular silique 19.3% (range: 1.9–43.8%), significantly lower than that of *pBjuA07*.*CLV1::BjuA07*.*CLV1* (t = 3.04, p < 0.01) (Fig. 4c,d). It suggests that the identified five non-synonymous SNPs in the CDS of *BjuA07*.*clv1* affected the number of carpels in multilocular siliques.

To further detect which of the five non-synonymous SNPs correlated with the changes of the of carpel number, the constructs of *pBjuA07*.*CLV1::BjuA07*.*clv1* with reverse mutation at position 83 (construct 3), 119 (construct 4), 151 (construct 5), 187 (construct 6) and 1014 (construct 7) in coding region, were transformed into *A*. *thalina* CS45 mutant, respectively, (Fig. 4b). The transgenic plants with the five constructs above were all showed paritial recovery of the bilocular silique (Fig. 4c). The average rescue rate (24.3%, 13.2% and 25.7%) of the transgenic plants with constructs 4, 5 and 7 showed no significant differences with that (19.3%) of the transgenic plants with construct 2 (constructs 4 vs 2: t = 0.531, p = 0.60; constructs 5 vs 2: t = 0.94, p = 0.36; constructs 7 vs 2: t = 1.005, p = 0.32) (Fig. 4c). However, the average rescue rate (43.6% and 38.0%) of the transgenic plants with construct 3 and 6, were significantly higher than that (19.3%) of the transgenic plants with construct 2 (constructs 3 vs 2: t = 2.98, p < 0.01; constructs 6 vs 2: t = 2.95, p < 0.01) (Fig. 4c). In addition, the average rescue rate of the transgenic plants with construct 3 and 6 showed no significant differences with that of the transgenic plants with construct 1 (constructs 3 vs 1: t = 0.19, p = 0.85; constructs 6 vs 1: t = 0.84, p = 0.41) (Fig. 4c). This suggests that SNP variations at position 83 and 187 in CDS (corresponding to the amino acid positions at 28 and 63) reduced the function of BjuA07.clv1 and increased the number of carpels in multilocular siliques.

### The 702-bp deletion in the promoter region of *BjuA07*.*clv1* affects the number of carpel

To confirm whether this 702-bp deletion influence promoter function, the *pBjuA07*.*clv1::BjuA07*.*CLV1* construct (Fig. 4b) was introduced to pCAMBIA2300, and transformed into *Arabidopsis* CS45 mutant. The transgenic plants of *pBjuA07*.*clv1::BjuA07*.*CLV1* showed some bilocular siliques (Fig. 4c,d). And the average rescue rate of the transgenic plants with the construct of *pBjuA07*.*clv1::BjuA07*.*CLV1* was 25.1%, which was significantly lower than that (45.5%) of the transgenic plants with construct of *pBjuA07*.*CLV1::BjuA07*.*CLV1* (t = 2.15, p < 0.05) (Fig. 4c,d). This suggests that the 702-bp deletion in the promoter region of *BjuA07*.*clv1* reduces the promoter function and affects the carpel number.

### *BjuA07*.*CLV1* and *BjuA07*.*clv1* exhibit significant differences in transcription level

**Figure 5:**
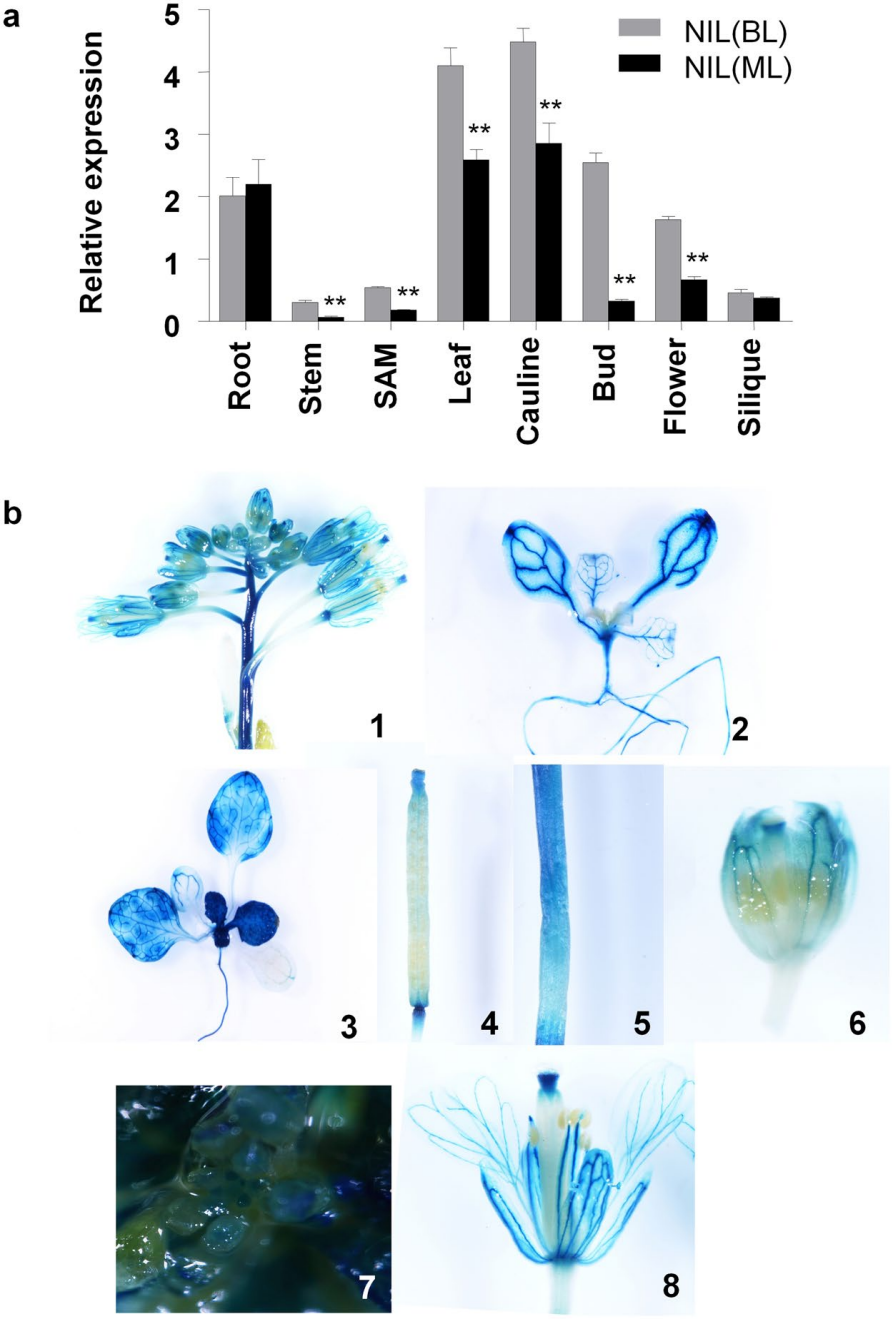
Expression pattern analysis of *BjuA07*.*CLV1* between bilocular and multilocular plants of near isogenic line using real time quantitative polymerase chain reaction (RT-qPCR) and β-glucuronidase (GUS) assay. (**a**) Comparison of BjuA07.CLV1 expression between NIL (BL) and NIL (ML) in various tissues by using qRT-PCR. The reference gene is β-actin. The values are the averages of three biological replicates; SDs are shown as error bars. **P < 0.01, and *P < 0.05. (**b**) GUS expression patterns (blue staining) in different tissues from the Pro_*BjuA07*_._*CLV1*_-GUS transgenic line; With the exception of siliques (4), GUS staining was detected in all tested tissues, including inflorescence (1), cotyledon (2), seedling (3), root (2,3), stem (5), bud (6), SAM (7) and flower (8).

Real-time quantitative PCR analysis revealed that *BjuA07*.*CLV1* was expressed in various organs including root, stem, SAM, leaf, cauline, bud, flower and silique (Fig. 5a). Further expression analysis using the Pro_*BjuA07*_._*CLV1*_-GUS fusion vector revealed that GUS staining was detected in different tissues (Fig. 5b), consistent with the results of qRT-PCR. However, the expression level of *BjuA07*.*clv1*was remarkably reduced in most tissues compared with that of *BjuA07*.*CLV1* (Fig. 5a). In stem and SAM, the expression of *BjuA07*.*clv1* decreased to approximately one-third of that of *BjuA07*.*CLV1*. And in buds, the expression level of *BjuA07*.*clv1* was reduced significantly to only one-seventh of that in bilocular plants (Fig. 5a). This suggests that the 702-bp deletion in the promoter region of *BjuA07*.*clv1* reduc its expression level, thereby cause the multilocular trait.

### BjuA07.CLV1 encodes a receptor kinase localised in the plasma membrane

**Figure 6:**
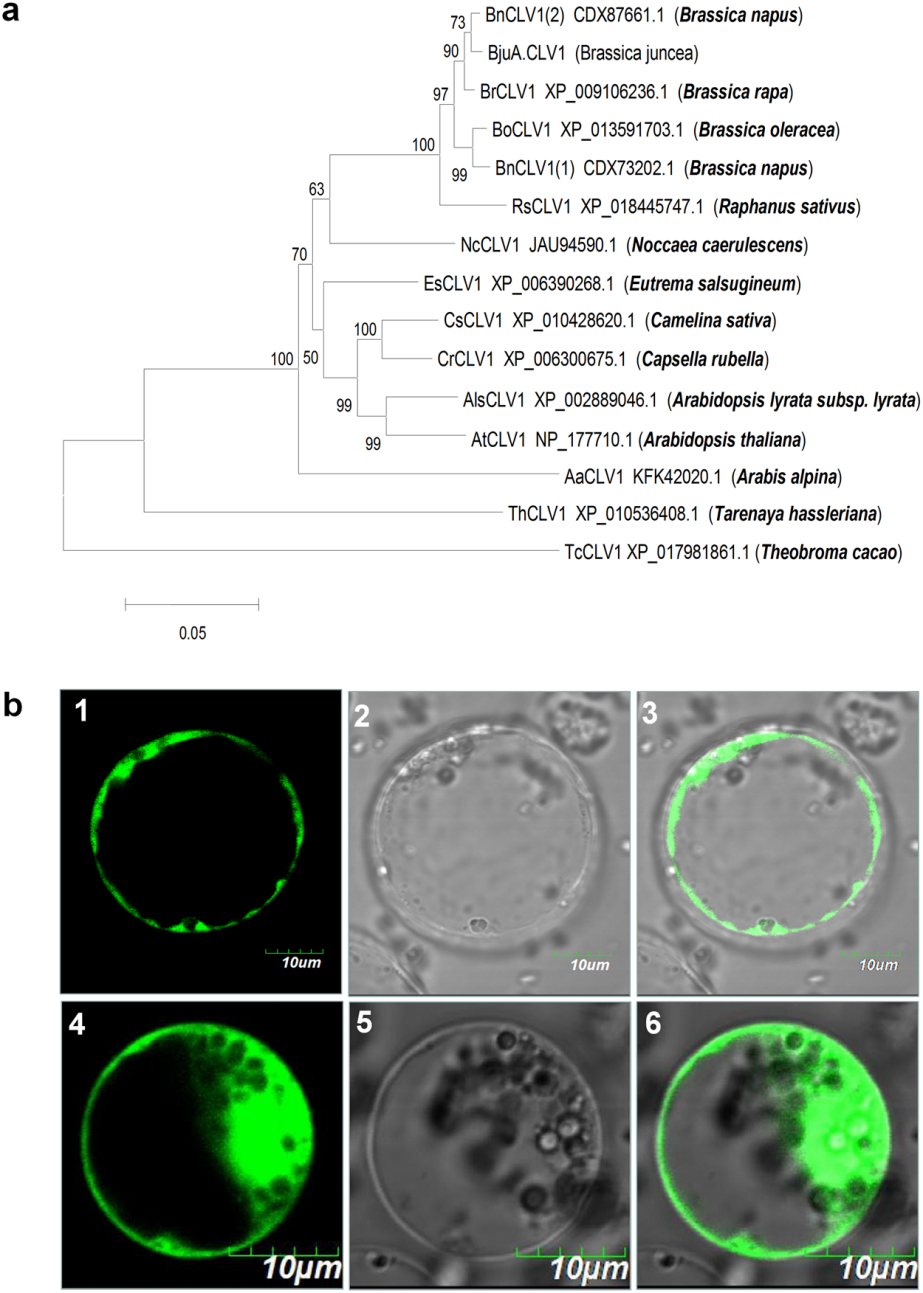
Sequence analysis of *BjuA07*.*CLV1* and its subcellular localisation. **(a)** MEGA4.0 was used for phylogenetic tree construction using the bootstrap neighbour-joining method. Proteins were designated according to the gene names of the accession numbers in NCBI. The branch lengths show variation rates of the amino acids. **(b)** Subcellular localisation of the BjuA07.CLV1-GFP fusion protein in Arabidopsis protoplasts. (1) The protoplast cell with BjuA07.CLV1-GFP fusion construct showed green fluorescent signal under a fluorescent filter; (2) The same cell under bright field; (3) The images in (1) and (2) were merged. Bars = 10 μM in (1–3); (4) The green fluorescent signal was detected throughout the protoplast cells with control GFP construct; (5) The same cell as (4) under bright field; (6) The images in (4) and (5) were merged. Bars = 10 μM in (4–6).

The full-length amino acid sequence of BjuA07.CLV1 was subsequently submitted to the NCBI for Blast analysis. The 14 closest homologues—i.e., one *B*. *rapa*, two *B*. *napus*, one *B*. *oleracea*, one *A*. *thaliana*, one *A*. *lyrata*, one *Raphanussativus*, one *Noccaeacaerulescens*, one *Camelinasativa*, one *Capsellarubella*, one *Arabisalpina*, one *Tarenayahassleriana*, one *Theobroma cacao*, and one *Eutremasalsugineum*—were further used to construct a phylogenetic tree. The deduced amino acid sequences of BjuA07.CLV1 had considerably higher sequence identity (99%) with the predicted amino acid sequences for one *B*. *napus* [BnCLV1 (2) and a *B*. *rapa* species (BrCLV1; Fig. 6a). The above results implied that BjuA07.CLV1 was considerably closer to BrCLV1 in evolution.

Sequence alignment further revealed that BjuA07.CLV1 exhibited higher sequence identity with *Arabidopsis* CLV1 (AtCLV1): the two sequences were more than 88% homologous (Fig. 6a). In *Arabidopsis*, the *clv1* mutant plant showed the multilocular phenotype^16^. The *At*.*CLV1* gene encoded a receptor-like kinase consisting of an extracellular leucine-rich repeat (LRR) domain, an intracellular kinase domain, and a transmembrane domain, thus implying that it plays a role in signal transduction^16^. To further verify whether BjuA07.CLV1 has the similar function as At.CLV1 in *Arabidopsis*, the subcellular localisation of BjuA07.CLV1 was investigated. Strong BjuA07.CLV1-GFP fluorescence was localised to the plasma membrane (Fig. 6b). It suggested that BjuA07.CLV1 could function as a receptor-like kinase during cell-to-cell signal transduction.

### *BjuA07*.*clv1* disturbed the *CLV/WUS* pathway

**Figure 7:**
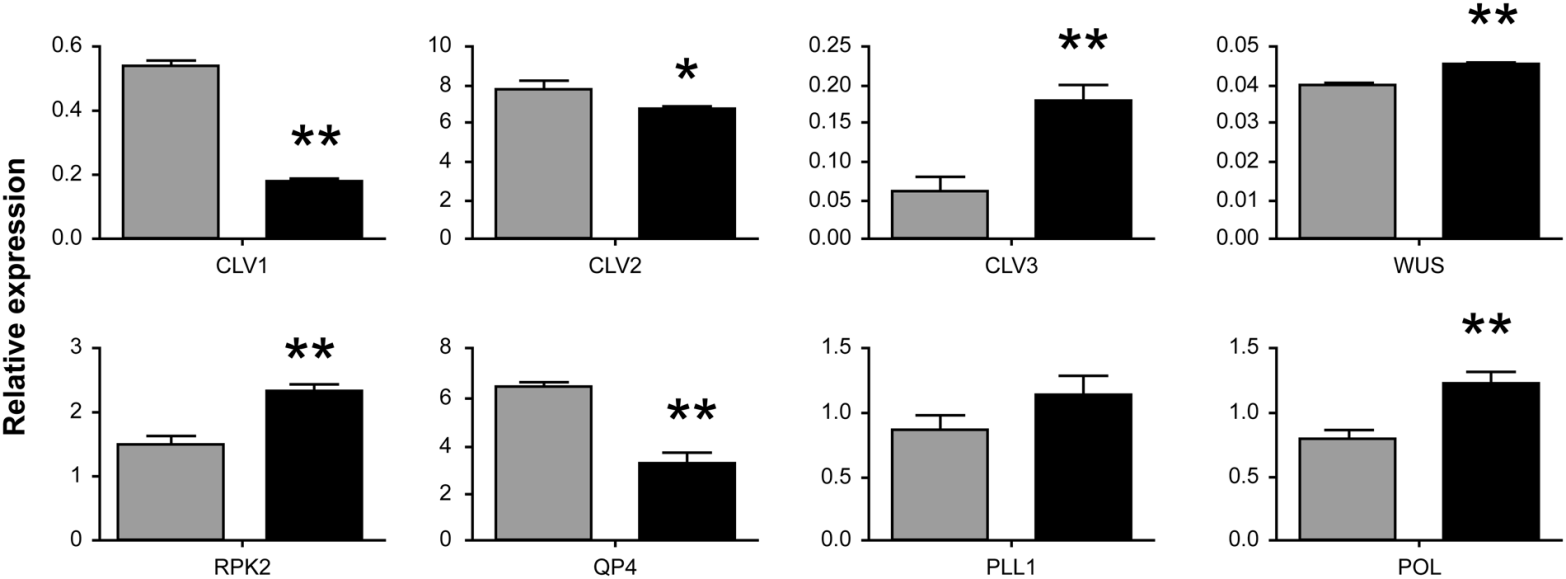
Real time quantitative polymerase chain reaction (RT-qPCR) analysis used for the detection of the expression level of *Arabidopsis* homologous genes participating in the *BjuA07*.*CLV1* signalling pathway in the shoot apical meristem (SAM) of the near-isogenic line (NIL) bilocular (gray column) and multilocular plants (black column). The values are the averages of three biological replicates; SDs are shown as error bars. β-actin was used as the reference. **P < 0.01 and *P < 0.05.

Further expression analysis of key genes involved in the *CLV*/*WUS* pathway was performed using the following *A*. *thaliana* homologues: *CLAVATA2* (*CLV2*), *CLAVATA3* (*CLV3*), *WUSCHEL* (*WUS*), *RECEPTOR-LIKE PROTEIN KINASE 2* (*RPK2*), *POLTERGEIST* (*POL*), and *PLL1*. Specific primers for these gene homologues were obtained from public sources^12^. Specific primers (qP-F and qP-R) for the *CLV1* homologue were designed based on the sequence of *BjuA07*.*CLV1* in *B*. *juncea*. The expression of the *WUS*, *CLV3*, *RPK2* and *POL* homologues increased in the SAM of near-isogenic lines (NILs) of multilocular plants compared with that of bilocular plants, however, *CLV2* expression decreased (Fig. 7). This suggests that the variation of *BjuA07*.*clv1* in multilocular plants disturbed the *CLV/WUS* signalling pathway.

## Discussion

In Brassicaceae, comparative genome analysis between *A*. *thaliana* and other *Brassica* species revealed significant similarity between the A and C and A and B genomes^15,18^. Recognition of the ancestral karyotype and presentation of the genomic block system reflect the conserved nature of crucifer genomes and might facilitate comparative genome studies between A. *thaliana* and *Brassica*^19^. Based on the genome synteny between A. *thaliana* and other *Brassica* species, researchers have successfully cloned and characterised economically important genes in *Brassica* plants without sequencing^20–22^. However, previous studies suggested that two reciprocal translocations, three chromosome fusions, and at least three inversions were instrumental in the derivation of *A*. *thaliana* from the ancestral karyotype, and evolutionary rearrangements led to karyotype diversification of one linkage group each between the A–B and A–C *Brassica* genomes^15,23,24^. Therefore, extensive chromosome segment duplication and chromosome rearrangements within synteny regions make it difficult to identify candidate genes in *Brassica* plants without sequencing^18,19^.

In this study, a positional cloning approach combined with the micro synteny that exists between the sequenced genomes (*A*. *thaliana* and *B*. *rapa*) and the unsequenced *B*. *juncea* genome were used to clone the *BjLn1* gene. Previous studies delimited the *BjLn1* gene to a 208-kb homologous interval on the A07 chromosome of *B*. *rapa*^14^. Blast analysis further revealed that the *BjLn1* flanking region has significant collinearity with *Arabidopsis* chromosome 1. Therefore, sequence information of putative *Arabidopsis* syntenic regions and the putative *B*. *rapa* homologous interval were used to develop IP and SSR markers. *BjLn1* was then delimited to an 85-Kb region. Previous studies suggested the existence of a high level of synteny between the A genomes of *B*. *rapa*, *B*. *napus*, and *B*. *juncea*^25,26^. In addition, high conservation at the macroscopic level was found between allotetraploid *B*. *juncea* and its diploid ancestors (*B*. *rapa* and *B*. *nigra*)^15^. In this study, the target region flanking *BjLn1* in *B*. *juncea* had significant synteny with the corresponding region in A07 of *B*. *rapa*. The candidate gene was then predicted and cloned according to the information on the syntenic gene in *A*. *thaliana* and *B*. *rapa* and validated by plant transformation.

Sequence comparison in the CDS between *BjuA07*.*CLV1* and *BjuA07*.*clv1* indicated that five SNPs in *BjuA07*.*clv1* caused amino acid changes. Further complementation test in *A*. *thaliana* CS45 mutant confirmed that amino acid changes at positions 28 and 63 were critical to the formation of multi-carpels. The domain architecture of BjuA07.CLV1 was predicted using NCBI and SMART (http://smart.embl-heidelberg.de/), which showed that BjuA07.CLV1, comprising the Leucine rich repeats domain (LRRs), transmembrane domain, and tyrosine kinase domain, was similar to that of CLV1 in *Arabidopsis*. The amino acid variations at positions 28 and 63 lie in the putative LRR domain. Previous studies have shown that LRRs are usually involved in protein–protein interactions. Each LRR consists of sections of β-strand and α-helix, and the protein forms a donut-shaped structure when combined with other repeats^16^. LRRs are responsible for the high-affinity combination of their corresponding ligands, leading to homodimerisation^16^. After oligomerisation, the cytoplasmic kinase domain might first autophosphorylate and then recruit specific downstream components. Therefore, two amino acid changes at positions 28 and 63 in the LRR domain in Duoshi would reduce the ability of ligand binding and further affect signal conversion in the SAM and FM, thereby causing the occurrence of larger SAM and multiple carpels.

Sequence analysis in the promoter region revealed that the *BjuA07*.*clv1*contained a 702-bp deletion 1,015 bp upstream of the start codon. Further transformations implied that the 702-bp deletion reduced promoter function and underlie its effect on multilocular phenotype. Search for cis-regulatory elements in the 702-bp region of *BjuA07*.*CLV1* was performed using PlantCARE (http://bioinformatics.psb.ugent.be/webtools/plantcare/html/). The 702-bp missing promoter region of *BjuA07*.*clv1* had 8 “CAAT box” and 1 “CAT” boxes. “CAAT box” is a common cis-acting element in the promoter and enhancer regions that can increase the transcript of a gene. And the “CAT box” is a cis-regulatory element related to meristem expression. Therefore, it could be inferred that the 702-bp deletion in the promoter region of *BjuA07*.*clv1* resulted in its reduced transcription level and caused multilocular trait.

The lower expression level of *BjuA07*.*clv1* (*CLV1* homologue) in SAM of NILs of multilocular plants might have caused the deregulation of *WUS* homologue, leading to an increase in its expression. In *Arabidopsis*, *WUS* is sufficient to induce the expression of *CLV3*. The *CLV3* homologue was highly expressed in multilocular plants. Previous studies indicated that *POLTERGEIST* (*POL*) acts downstream of the CLV proteins: CLV signalling represses POL, which is required for WUS expression^27^. The expression level of the *POL* homologue was elevated in the NILs of multilocular plants. Taken together, these findings suggest that lower *BjuA07*.*clv1* expression disturbed the *CLV*/*WUS* feedback loop, leading to an enlarged SAM that generates multilocular phenotype. In *Arabidopsis*, *BAM1* and *BAM2* seem to play contrary development roles to *CLV1* in the meristem^10^. In the present study, the expression level of *BAM1* homologue showed no significant differences between the SAM of multilocular (ML) and bilocular (BL) NIL plants. Similar to *BAM1*, *BAM2* homologue expression in the SAM of NIL (ML) was not obviously different from that of NIL (BL) (data not shown).

The SAM in higher plants is the source for the development of above ground organs and tissues by facilitating constant organogenesis via the renewal of a pool of cells at the centre of the meristem and conducting offspring to organ differentiation at the peripheral zone^16,28^. It maintains almost the same dimension throughout a plant’s life cycle while the resident cells constantly renew, implying a balance between the division rate of the undifferentiated cells in the CZ and that of the differentiated cells in the PZ^16^. In *B*. *juncea*, the enlargement of SAM in NIL (ML) was first observed in the early stage of inflorescence meristem differentiation; however, in the vegetative period, no significant difference was detected in the size of SAM between NILs of ML and BL. In addition, the diameter of FM in NIL (ML) was significantly larger than that in NIL (BL). Increased FM size leads to the formation of multi-carpels and endogenous carpel in the medical regions of the developing gynoecia. These results suggested that *BjuA07*.*CLV1* is required for both meristem and gynoecium development. Moreover, the presence of multiple carpels results in the occurrence of multiple seed rows, and multiple carpels and endogenous gynoecium contribute to the increase in the number of seeds per silique in plants, which is of great significance for the improvement of yield in *B*. *juncea*.

## Materials and Methods

### Plant materials and population development

The plants used for mapping were cultivated in a field under naturalistic conditions. Transgenic plants were cultivated in growth chambers with a 16-h photoperiod, with temperatures of 24 °C and 16 °C for day and night, respectively. The *Arabidopsis* CS45 mutant was purchased from the ABRC (http://abrc.osu.edu/).

A BC3 population (4,387 individuals) was reconstructed in this study for mapping the *BjLn1* gene. This population was generated by crossing the bilocular line, Tayou2, with the multilocular line, Duoshi, and then backcrossing to Duoshi for three generations, as previously described^14^. The genotypes of the multilocular and bilocular plants in the BC3 population were *Bjln1Bjln1Bjln2Bjln2* and *BjLn1Bjln1Bjln2Bjln2*, respectively.

In the resulting BC3 population, a bilocular plant was randomly selected and self-pollinated. Next, 12 bilocular individuals were randomly selected from the resultant BC3F2 population and then self-pollinated. Each BC3F3 population that consisted entirely of bilocular plants was successively maintained by self-pollination for two generations, producing BC3F5 plants homozygous for *BjLn1*. The multilocular plants of BC3F2 were successively self-pollinated for three generations, leading to the development of BC3F5 plants homozygous for *Bjln1*. The homozygous bilocular and multilocular plants from BC3F5 that were NILs were used for phenotypic analysis and real-time quantitative PCR studies.

### Mapping of *BjLn1*

IP primers were designed according to the DNA sequence of the exon from the *Arabidopsis* gene in the homologous region. IP primers designation, amplification and analysis were performed as described by Panjabi *et al*.^15^. Next, sequences of the target region in *B*. *rapa* (http://brassicadb.org/brad/) were applied for SSR marker development. DNA extraction, IP and SSR marker analysis, sequencing, and mapping were carried out as previously described^21,26^. Blast analysis was used to map the SSR and IP sequences in the *Arabidopsis* and *B*. *rapa* genomes. Sequence alignment was performed as described by Dun *et al*.^21^.

### Vector construction and plant transformation

According to the DNA sequence of *clv1* orthologue in *B*. *rapa*, primers (FL-F and FL-R; Supplementary Table S1) were designed to amplify the corresponding sequences from Duoshi and Tayou2. PCR amplification was performed using Phusion^®^ Hot Star High Fidelity DNA polymerase (NEB, Ipswich, USA), and appropriate restriction enzyme cleavage sites were added. The fragment containing the *BjLn1* gene (two-locule-number allele), including the 5′(1,945 bp) and 3′(376 bp) ends, was gel isolated using the primers FL-F and FL-R and then introduced into the pCAMBIA2300 vector. Recombinants were cloned into *Escherichia coli TOP10* (Invitrogen, USA). Positive clones were selected using PCR, and the inserts were confirmed by sequencing. The verified recombinants were then mobilised into *Agrobacterium tumefaciens* GV3101. *Agrobacterium*-mediated transformation and regeneration of Duoshi was performed as previously described^21,29^. PCR analysis was used to screen the positive transformants by using primers M13 (−47) (from the vector) and TF (from the genomic sequence of the *BjLn1* gene; Supplementary Table S2). DNA of the T1 segregants was isolated from the leaves for cosegregation analysis by using the primers TF and M13 (−47). The carpel number of each transgenic plant was visually evaluated during the maturation stage.

The constructs of *pBjuA07*.*CLV1::BjuA07*.*CLV1* and *pBjuA07*.*CLV1::BjuA07*.*clv1*, the constructs of *pBjuA07*.*CLV1::BjuA07*.*clv1* with reverse mutation at position 83, 119, 151, 187 and 1014 in coding region, and construct of *pBjuA07*.*clv1::BjuA07*.*CLV1* were synthesized and then introduced into pCAMBIA2300 vector by GENEWIZ Inc. (China), respectively. All the recombinants were cloned into *Escherichia coli TOP10* (Invitrogen, USA), respectively. Positive clones were selected by PCR and sequencing-verified. All the resulting recombinants were transformed into *Agrobacterium tumefaciens* GV3101 and used to transform *A*. *thaliana* mutant CS45 by floral dip method^30^. The transgenic plants were screened and confirmed by using the primers TF and M13 (−47).

### Light microscopy

The same growth conditions were ensured by cultivating the homozygous bilocular and multilocular individuals from BC3F5 in adjacent lines in a greenhouse. The shoot apices of the seedlings with two leaves were excised under a dissecting microscope. The stem meristem size during various developmental stages was measured by sampling plants every 2 days. Samples were directly blended with formalin–acetic acid–ethanol (formalin, 5 ml; acetic acid, 5 ml; 50% ethanol, 90 ml) for 24 h at 4 °C. After the samples were dehydrated in a graded alcohol series (70%, 83%, 95%, and 100%) and processed through a series of chloroform–ethanol from 25% to 100%, the specimens were embedded in paraffin. A microtome (Leica RM2235) was used to cut the specimens into 3-μm-thick sections, which were subsequently stained with safranine and then imaged under a microscope.

### Scanning electron microscopy analysis

Fresh inflorescence meristems of homozygous bilocular and multilocular individuals from BC3F5 from different developmental stages were dissected and blended overnight in 2% glutaraldehyde in 0.1 M phosphate buffer saline solution (PBS, pH = 7.4) following incubation in osmium tetroxide in 0.1 M PBS. Next, they were dehydrated in a series of gradient ethanol solutions (30%, 50%, 70%, 85%, 95%, and 100%). After the samples were dried using the critical point drying method, they were mounted on stubs by using double-sided tape and sputter-coated with gold, as previously described^31^, and examined using a JSM-6610/LV scanning electronic microscope.

### cDNA isolation and real-time quantitative PCR

The multilocular plants and homozygous bilocular plants in the BC3F5 generation were used for the *BjuA07*.*CLV1* gene expression studies. Plant materials were grown in adjacent lines in a greenhouse. Various tissues from multilocular and bilocular plants, including roots, stems, leaves, cauline leaves, buds, flowers, SAMs, and siliques, were collected from 3 plants with the same genotype and immediately frozen in liquid nitrogen.

Frozen tissues were ground to a fine power in liquid nitrogen, and total RNA was isolated using the EASYspin Plant RNA kit (Biomed, Beijing, China). After the samples were diluted 10-fold, the concentration of total RNA from each sample was determined using a spectrophotometer (Bio Spectrometer Basic, Eppendorf). Total RNA (5 μg) was used to synthesise the first-strand cDNA with the PrimeScript™ RT reagent kit using gDNA Eraser (Takara, Dalian). The q-PCR amplification was performed using specific primers for the *BjuA07*.*CLV1* gene, i.e., qP-F and qP-R (Supplemental Table S1). The β-actin (BnaC02g00690D) gene, used as a control, was amplified using the primers β-actin F and β-actin R (Supplementary Table S1).

### The β-glucuronidase assay

The genomic region corresponding to approximately 1.9 Kb upstream of the *BjuA07*.*CLV1* gene were amplified from the homozygous bilocular plants by using primers Pro-F and Pro-R (Supplementary Table S1), respectively. The resulting fragment was cloned into the plant transformation vector PBI101 and then introduced into the wild-type *Arabidopsis* ecotype Col-*0* by using *Agrobacterium*-mediated genetic transformation. Samples were prefixed in 90% acetone for 20 min on ice. After they were rinsed with wash buffer (50 mM NaPO_4_), the tissues were kept in X-Gluc solution (10 mM NaPO_4_, pH 7.2; 5 mM potassium ferrocyanide; 5 mM potassium ferricyanide; 10 mM X-gluc) overnight^32^. The tissues were incubated at 37 °C for 24 h and then cleaned in 70% alcohol. The treated tissues were imaged under a microscope as previously described^21,33^.

### Subcellular localisation

The cellular localisation of BjuA07.CLV1 was investigated by cloning the entire BjuA07.CLV1 coding region from Tayou2 without a stop codon in frame by PCR amplification by using special primers PB-F and PB-R (Supplementary Table S1), and then introducing them into the pM999GFP vector. The fusion construct of 35 S BjuA07.CLV1-GFP was subsequently introduced into *Arabidopsis* protoplasts by PEG/calcium-mediated transformation^34^. The fluorescence signals were detected and imaged using confocal microscopy and visualized using Olympus Microsystem FV10-ASW.

### Computational analyses

The BjuA07.CLV1 amino acid sequence was used for searching homologues using the BLASTP program of the National Centre for Biotechnology Information (http://www.ncbi.nlm.nih.gov/). MEGA software (version 4.0) (http://www.megasoftware.net/index.html) was used for phylogenetic tree construction. MUSCLE was used for protein sequence alignment^35^.

## Electronic supplementary material

Supplementary material
