# Supplementary material for: Mutations in the CDS and promoter of BjuA07.CLV1 cause a multilocular trait in Brassica juncea
Source: Sci Rep. 2018 Mar 28;8:5339. doi: 10.1038/s41598-018-23636-4 (PMC5871799; doi:10.1038/s41598-018-23636-4)

Mutations in the CDS and promoter of *BjuA07.CLV1* cause a multilocular trait in *Brassica juncea*

Lu Xiao[1]†, Xin Li[1]†, Fei Liu[2], Zhi Zhao[1], Liang Xu[1], Cuiping Chen[1], Yanhua Wang[1], Guoxia Shang[1], Dezhi Du[1]*

Supplementary Table S1 Primer sequences of molecular markers developed in this study

Marker	Sequence (5′-3′)
ln10 F	GCGCTAGACTTCCTTGTGTG
ln10 R	CCTTAGCCTCTGTGTCTTCG
ln11 F	GCAAGTCTGATCGTAGCGTC
ln11 R	TTCACCTCCAAGAGAGAACC
ln12 F	GCTTCTGTACTGCTTCCACC
ln12 R	ATAGCCACCGAGTCGAATTG
ln13 F	ATCGTAAGCCATCCATCCTC
ln13 R	ATACCACCAGGCAACATGAG
ln14 F	CATGATCCATGTTGTGCTCC
ln14 R	TCTCTTCGCTGCTCTTGTTG
ln15 F	ACGCTTCCATTGTCCAGAAC
ln15 R	GCGACGTGGTACGGATTATG
ln16 F	GCATACGATCAGCGATAGTG
ln16 R	TAGACCTTCTTCTGCTGTGC
FL-F	CTCCAGATCATCATCATCAT
FL-R	CACAGCAGAAGAAGGTCTAC
qP-F	CGGCGAATTGACAAACCTAGAGGT
qP-R	AGGACTTGGAGGTTCGGCATGT
β-actin F	GAGCTCCGTGTTGCCCCTGAAGA
β-actin R	TGGATAGCAACATACATGGCAGGGACA
pro-F	CCCAAGCTTCAGCAGAAGAAGGTCTACAA
pro-R	TCCCCCGGGAAAAGCTGTCTTTTGAGAGA
M13 (-47)	CGCCAGGGTTTTCCCAGTCACGAC
TF	CCGGAATTCAGTTAGACTCCTCGGATACG
PB-F	TACtctagaATGTACGTCGGCTACTTCAACAGCTAC
PB-R	TACtctagaGGTTAGGTTGTTAGCACTCGTGTTGAT

Supplementary Fig.S1 Frequency distribution with respect to bilocular silique percentage in untransformed control (the left column) and 25 T0 plants (the second to the last column) carrying the candidate gene.

Supplementary Fig.S2 The examination of insertion fragment and genetic background of some T1 individuals derived from one of the T0 plants. (a) The examination of insertion fragment by the specific primer TF and M13 (-47). Lanes 1-4 represent the non-transgenic multilocular plants (negative control), lanes 5-6 represent the non-transgenic bilocular plants (positive control), lanes 7-38 represent the bilocular plants in transgenic T1 progenies, lanes 39-48 represent the multilocular plants in T1 progenies, M represent the DNA ladder, ladders from top to bottom are 2000bp, 1000bp, 750bp, 500bp, 250bp and 100bp. (b) The examination of genetic background using molecular marker IP18. Lanes 1-4 represent the non-transgenic bilocular plants (positive control), lanes 5-36 represent the bilocular plant in transgenic T1 progenies, lanes 37-46 represent the multilocular plants in T1 progenies, M 100-bp DNA ladder.

Supplementary Fig.S3 Comparison of the BjuA07.CLV1 and BjuA07.clv1 coding sequences (c) and genomic sequences (g). The coding sequences of BjuA07.CLV1 and BjuA07.clv1 were isolated by cDNA sequencing by using RNA from buds of NIL (BL) and NIL (ML) in BC3F5. Sequences were aligned using GeneDoc. Black boxes and gray ones indicate identical residues and similar residues, respectively.

Supplementary Fig.S4 Amino acid sequence comparison of BjuA07.CLV1 from Duoshi and BjuA07.clv1 from Tayou2. GeneDoc was used for sequence alignment. Identical and similar residues are shown as black and grey boxes, respectively.

Supplementary Fig. S5 Comparison of a putative upstream promoter region between the BjuA07.CLV1 of Tayou2 and BjuA07.clv1 of Duoshi. GeneDoc was

used for sequence alignment. Black boxes and gray ones indicate identical residues and similar residues, respectively.

Figure S1

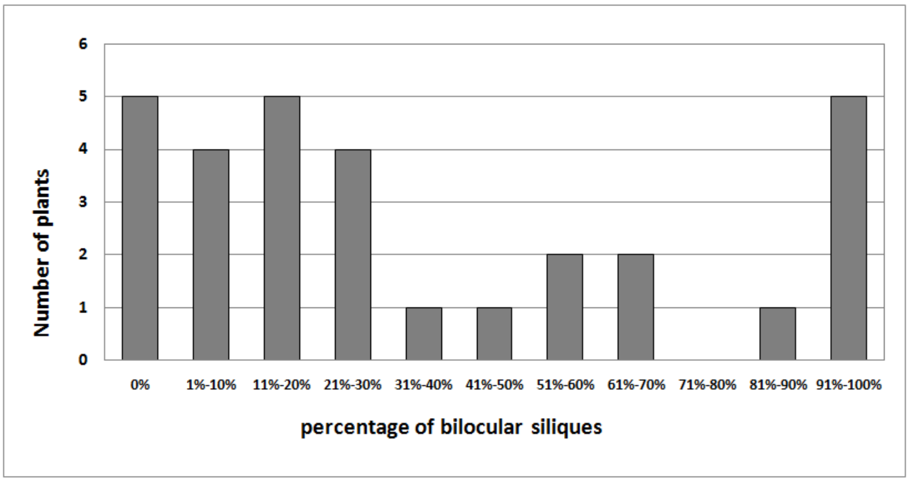

6
5
4
3
2
1
0
Number of plants
0%
1%-10%
11%-20%
21%-30%
31%-40%
41%-50%
51%-60%
61%-70%
71%-80%
81%-90%
91%-100%
percentage of bilocular siliques

Figure S2

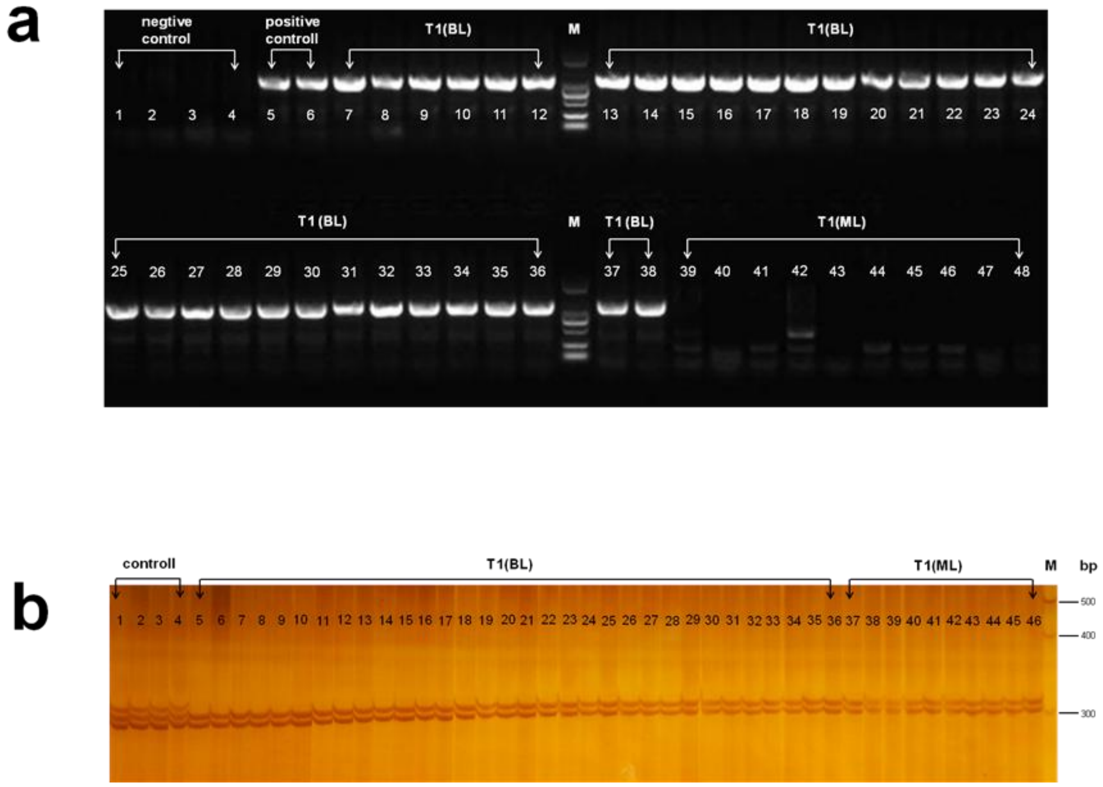
a
negtive control
positive controll
T1(BL)
M
T1(BL)
1 2 3 4 5 6 7 8 9 10 11 12 13 14 15 16 17 18 19 20 21 22 23 24
T1 (BL)
M
T1 (BL)
T1(ML)
25 26 27 28 29 30 31 32 33 34 35 36 37 38 39 40 41 42 43 44 45 46 47 48
b
controll
T1(BL)
T1(ML)
M
bp
1 2 3 4 5 6 7 8 9 10 11 12 13 14 15 16 17 18 19 20 21 22 23 24 25 26 27 28 29 30 31 32 33 34 35 36 37 38 39 40 41 42 43 44 45 46
500
400
300

Figure S3

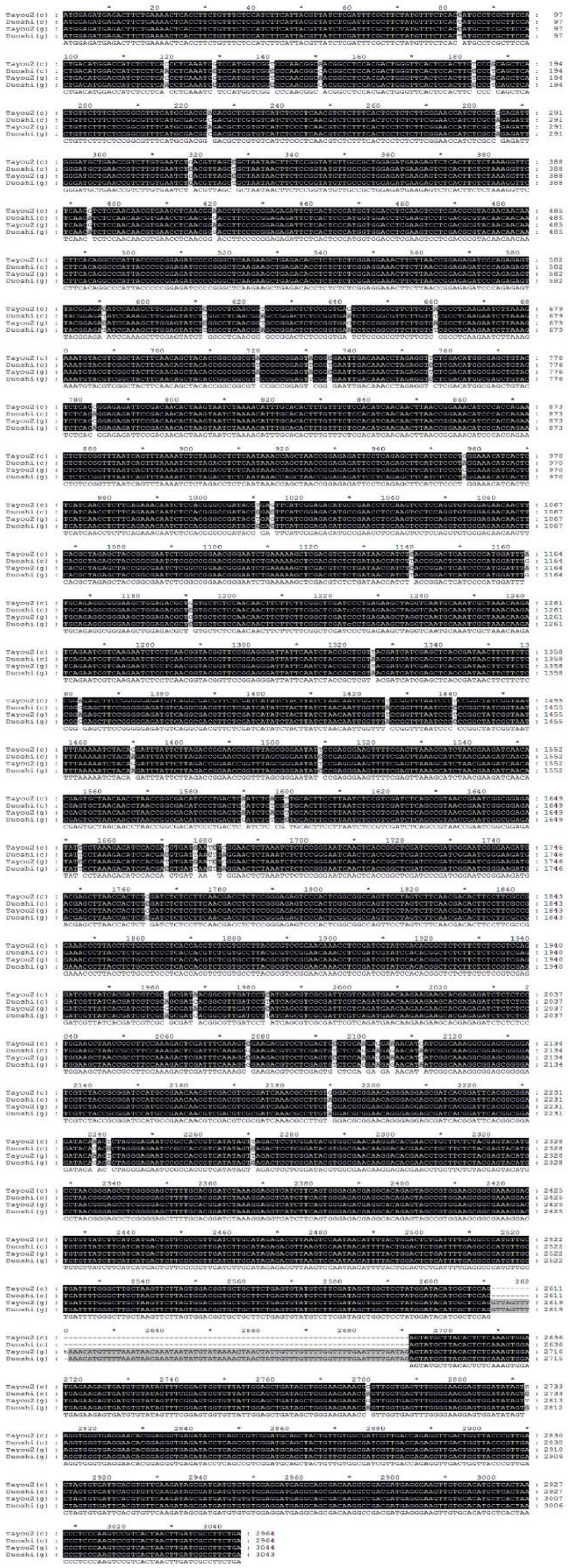

Figure S4

```
                  *        20         *        40         *        60         *        80         *       100
Tayou2 : MEMRLLKTHLLFLHLHYVISISLLCFSPCLASTDMDHLLTLKSSMVGPNGNGLHDWVHSTSPAAHCSFSGVSCDGDARVISLNVSFTPLFGTISPEIGMLN : 101
Duoshi : MEMRLLKTHLLFLHLHYVISISLLCFSLCLASTDMDHLLNLKSSMVGPNGHGLHDWVHSTSPSAHCSFSGVSCDGDARVISLNVSFTPLFGTISPEIGMLN : 101
         MEMRLLKTHLLFLHLHYVISISLLCFS CLASTDMDHLL LKSSMVGPNG GLHDWVHSTSP AHCSFSGVSCDGDARVISLNVSFTPLFGTISPEIGMLN

                 *       120         *       140         *       160         *       180         *       200
Tayou2 : RLVNLTLAANNFSGMLPLEMKSLTSLKVLNVSNNVNLNGTFPGEILTPMVDLEVLDAYNNNFTGPLPPEIPGLKKLRHLSLGGNFLTGEIPESYGDIQSLE : 202
Duoshi : RLVNLTLAANNFSGMLPLEMKSLTSLKVLNISNNVNLNGTFPGEILTPMVDLEVLDAYNNNFTGPLPPEIPGLKKLRHLSLGGNFLTGEIPESYGDIQSLE : 202
         RLVNLTLAANNFSGMLPLEMKSLTSLKVLN6SNNVNLNGTFPGEILTPMVDLEVLDAYNNNFTGPLPPEIPGLKKLRHLSLGGNFLTGEIPESYGDIQSLE

                *       220         *       240         *       260         *       280         *       300
Tayou2 : YLGLNGAGLSGESPAFLSRLKNLKEMYVGYFNSYTGGVPPEYGELTNLEVLDMASCTLTGEIPTTLSNLKHLHTLFLHINNLTGNIPPELSGLISLKSLDL : 303
Duoshi : YLGLNGAGLSGESPAFLSRLKNLKEMYVGYFNSYTGGVPPEFGELTNLEVLDMASCTLTGEIPTTLSNLKHLHTLFLHINNLTGNIPPELSGLISLKSLDL : 303
         YLGLNGAGLSGESPAFLSRLKNLKEMYVGYFNSYTGGVPPE5GELTNLEVLDMASCTLTGEIPTTLSNLKHLHTLFLHINNLTGNIPPELSGLISLKSLDL

               *       320         *       340         *       360         *       380         *       400
Tayou2 : SINQLTGEIPQSFISLGNITLINLFRNNLHGPIPEFIGDMPNLQVLQVWENNFTLELPANLGRNGNLKKLDVSDNHLTGLIPMDLCRGGKLETLVLSNNFF : 404
Duoshi : SINQLTGEIPQSFISLGNITLINLFRNNLHGPIPDFIGDMPNLQVLQVWENNFTLELPANLGRNGNLKKLDVSDNHLTGLIPMDLCRGGKLETLVLSNNFF : 404
         SINQLTGEIPQSFISLGNITLINLFRNNLHGPIP FIGDMPNLQVLQVWENNFTLELPANLGRNGNLKKLDVSDNHLTGLIPMDLCRGGKLETLVLSNNFF

              *       420         *       440         *       460         *       480         *       500
Tayou2 : FGSIPEKLGQCKSLNKIRIVKNLLNGTVPEGLFNLPLVTIIELTDNFFSGELPGEMSGDVLDHIYLSNNWFSGLIPPAIGNFKNLQDLFLDRNRFSGNIPR : 505
Duoshi : FGSIPEKLGQCKSLNKIRIVKNLLNGTVPEGLFNLPLVTIIELTDNFFSGELPGEMSGDVLDHIYLSNNWFTGLIPPAIGNFKNLQDLFLDRNRFSGNIPR : 505
         FGSIPEKLGQCKSLNKIRIVKNLLNGTVPEGLFNLPLVTIIELTDNFFSGELPGEMSGDVLDHIYLSNNWF3GLIPPAIGNFKNLQDLFLDRNRFSGNIPR

             *       520         *       540         *       560         *       580         *       600
Tayou2 : EVFELKHLTKINTSANNLTGDIPDSISRCTSLISVDLSRNRIGGDIPKDIHDVMNLGTLNLSGNQLTGSIPIGIGKMTSLTTLDLSFNDLSGRVPLGGQFL : 606
Duoshi : EVFELKHLTKINTSANNLTGDIPDSISRCTSLISVDLSRNRIGGDIPKDIHDVINLGTLNLSGNQLTGSIPIGIGKMTSLTTLDLSFNDLSGRVPLGGQFL : 606
         EVFELKHLTKINTSANNLTGDIPDSISRCTSLISVDLSRNRIGGDIPKDIHDV6NLGTLNLSGNQLTGSIPIGIGKMTSLTTLDLSFNDLSGRVPLGGQFL

            *       620         *       640         *       660         *       680         *       700
Tayou2 : VFNDTSFAGNPYLCLPHHVSCLTRPEQTSDRIHTALFSPSRIVITIVAAITALILISVAIRQMNKKKHERSLSWKLTAFQRLDFKAEDVLECLQEENIIGK : 707
Duoshi : VFNDTSFAGNPYLCLPHHVSCLTRPEQTSDRIHTALFSPSRIVITIVAAITALILISVAIRQMNKKKHERSLSWKLTAFQRLDFKAEDVLECLQEENIIGK : 707
         VFNDTSFAGNPYLCLPHHVSCLTRPEQTSDRIHTALFSPSRIVITIVAAITALILISVAIRQMNKKKHERSLSWKLTAFQRLDFKAEDVLECLQEENIIGK

           *       720         *       740         *       760         *       780         *       800
Tayou2 : GGAGIVYRGSMPNNVDVAIKRLVGRGTGRSDHGFTAEIQTLGRIRHRHIVRLLGYVANKDTNLLLYEYMPNGSLGELLHGSKGGHLQWETRHRVAVEAAKG : 808
Duoshi : GGAGIVYRGSMPNNVDVAIKRLVGRGTGRSDHGFTAEIQTLGRIRHRHIVRLLGYVANKDTNLLLYEYMPNGSLGELLHGSKGGHLQWETRHRVAVEAAKG : 808
         GGAGIVYRGSMPNNVDVAIKRLVGRGTGRSDHGFTAEIQTLGRIRHRHIVRLLGYVANKDTNLLLYEYMPNGSLGELLHGSKGGHLQWETRHRVAVEAAKG

          *       820         *       840         *       860         *       880         *       900
Tayou2 : LCYLHHDCSPLILHRDVKSNNILLDSDFEAHVADFGLAKFLVDGAASECMSSIAGSYGYIAPEYAYTLKVDEKSDVYSFGVVLLELIAGKKPVGEFGEGVD : 909
Duoshi : LCYLHHDCSPLILHRDVKSNNILLDSDFEAHVADFGLAKFLVDGAASECMSSIAGSYGYIAPEYAYTLKVDEKSDVYSFGVVLLELIAGKKPVGEFGEGVD : 909
         LCYLHHDCSPLILHRDVKSNNILLDSDFEAHVADFGLAKFLVDGAASECMSSIAGSYGYIAPEYAYTLKVDEKSDVYSFGVVLLELIAGKKPVGEFGEGVD

         *       920         *       940         *       960         *       980
Tayou2 : IVRWVRNTEGEIPQPSDAATVVAIVDQRLTGYPLTSVIHVFKIAMMCVEDEAATRPTMREVVHMLTNPPKSVTNLIAF* : 987
Duoshi : IVRWVRNTEGEIPQPSDAATVVAIVDQRLTGYPLTSVIHVFKIAMMCVEDEAATRPTMREVVHMLTNPPKSVTNLIAF* : 987
         IVRWVRNTEGEIPQPSDAATVVAIVDQRLTGYPLTSVIHVFKIAMMCVEDEAATRPTMREVVHMLTNPPKSVTNLIAF
```

Figure S5

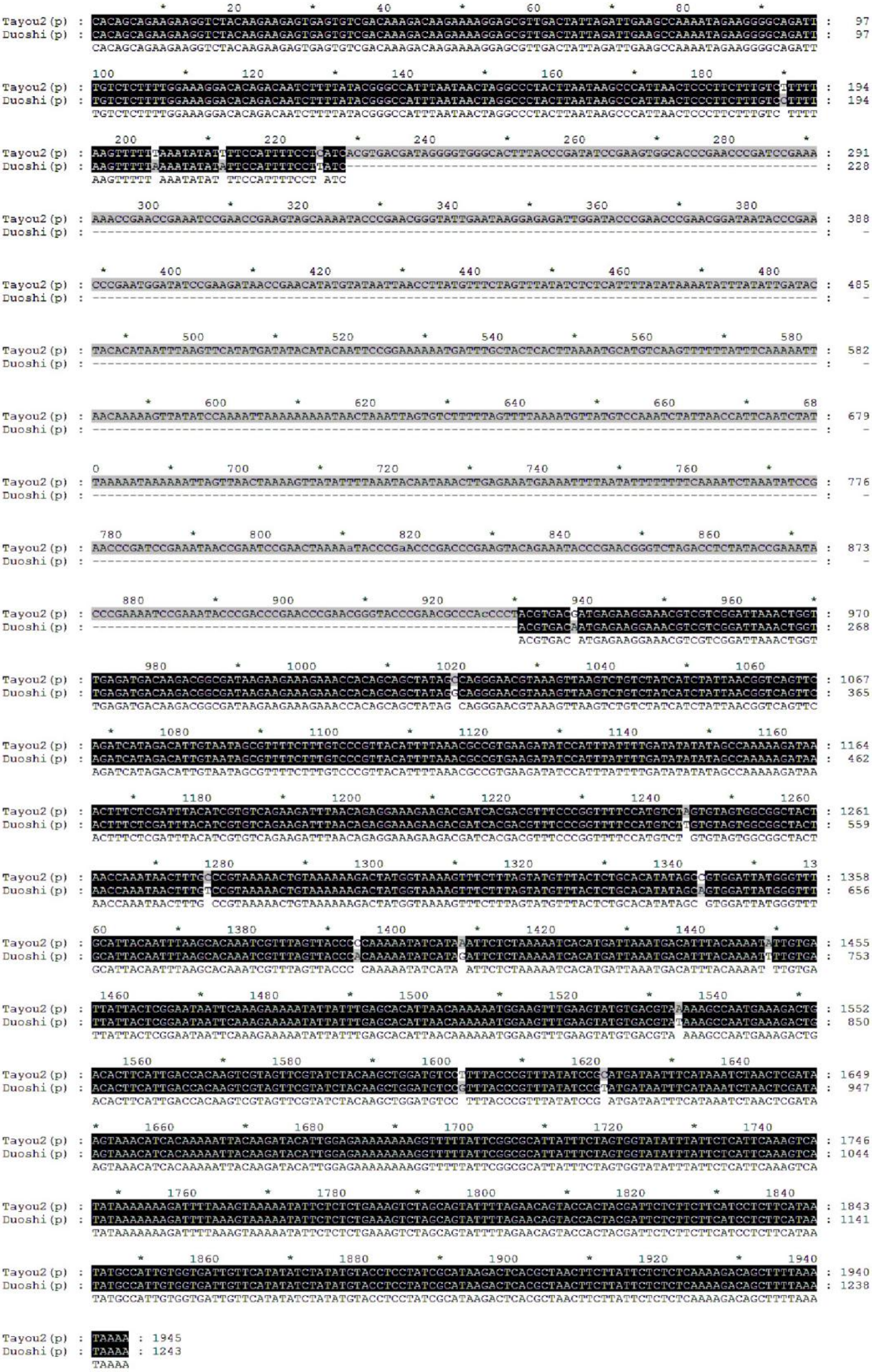

Supplement: Supplementary file 1 — Supplementary material [file 41598_2018_23636_MOESM1_ESM.pdf]
